# Rising Temperature Effects on Growth and Gastric Emptying Time of Freshwater African Catfish (*Clarias Gariepinus*) Fingerlings

**DOI:** 10.3390/ani11123497

**Published:** 2021-12-08

**Authors:** Sonia Mohd Kasihmuddin, Mazlan Abd. Ghaffar, Simon Kumar Das

**Affiliations:** 1 Department of Earth Sciences and Environment, Faculty of Science and Technology, National University of Malaysia, Bangi 43600, Selangor, Malaysia; P109937@siswa.ukm.edu.my; 2 Climate Change Adaptation Laboratory, Institute of Marine Biotechnology, Universiti Malaysia Terengganu, Kuala Nerus 21030, Terengganu, Malaysia; mag@umt.edu.my; 3 Higher Institution Centre of Excellence (HICoE), Institute of Tropical Aquaculture and Fisheries (AKUATROP), Universiti Malaysia Terengganu, Kuala Nerus 21030, Terengganu, Malaysia; 4 Marine Ecosystem Research Centre (EKOMAR), Faculty of Science and Technology, National University of Malaysia, Bangi 43600, Selangor, Malaysia

**Keywords:** Clariidae, freshwater fish, fish physiology, digestion, aquaculture

## Abstract

**Simple Summary:**

Fish are influenced by their surroundings. We investigated the effect of water temperature ranging from 26 °C to 32 °C on the growth performance and gastric emptying time (GET) of commonly cultured African catfish *Clarias gariepinus* fingerlings. Water temperatures between 26 °C and 32 °C were satisfactory for the growth of African catfish fingerlings, with GET observed between 10 and 16 h. Our experiment provides baseline data of the effects of water temperature on the culture of catfish.

**Abstract:**

The present study was carried out to analyse the effect of water temperature on two components: (1) growth performance, and (2) gastric emptying time (GET) of African catfish *Clarias gariepinus* fingerlings. After 70 days, it was observed that experimental temperatures had no significant effects on the growth performance parameters, except for food conversion ratio (FCR) and food conversion efficiency (FCE). GET observation through X-radiography denoted that the shortest GET (10 h) was observed in fish reared at 32 °C and the longest GET (16 h) was observed in fish reared at 26 °C. The rapid digestion rate coincides with the FCR and FCE obtained in this study. Considering the limited scope of our study, more extensive studies on the impact of water temperature on other fish physiological parameters should be pursued. A better understanding of this research topic would be beneficial for the growth of African catfish fingerling aquaculture.

## 1. Introduction

African catfish *Clarias gariepinus*, classified under *Clarias* spp., is among the most cultured freshwater finfish species in the world [1]. In major countries of production, this fish has a steadily developing market value since the demands for fish to cater to population needs are constantly increasing [2]. This introduced freshwater fish species is also favoured by fish farmers due to its rapid growth rate [3], high tolerance to water quality [4], and huge diversification of culture environment, from extensive traditional ponds to intensive recirculating aquaculture system (RAS) tanks [2]. 

Physical factors such as temperature are of great importance in the life cycle of poikilothermic species [5]. This abiotic master factor could control fish growth [6] as well as their overall physiological activity [7,8]. In general, fish growth increases with increasing water temperature up to a certain point but declines abruptly once the critical limit has been reached [9]; this is due to the increase in energy cost to maintain their metabolism [10]. The optimal growth of fish often lies within their preferred water temperature [11]. 

Over the past decades, the best temperature for rearing African catfish has been well established; around 30 °C [12]. However, no information on the impact on specific physiological components such as gastric emptying time (GET) has been recorded. Determination of GET is critical based on the assumption that the duration of time for which food items are entirely removed from the stomach is equal to the rate of food completely digested [13]. This physiological component is crucial to estimate the fish feeding rates, energy budgets, and daily rations [10,14,15].

The aim of this study was to determine the effects of water temperature on both the growth performance and GET of African catfish. In this study, temperature selection was based on the fundamental thermal niche (FTN) of species, whereby the “minus three and plus one” formula [16] was applied to the suggested temperature for the optimal growth of African catfish (~30 °C) [12]. By diving deeper into this study, it will surely bring profitable results, meeting with the ever-increasing demands for fish and fish products [2,17] through the most effective aquaculture techniques.

## 2. Materials and Methods

### 2.1. Fish Collection and Experimental Design 

Fingerlings of *C. gariepinus* (*n* = 200, body weight = 15.0 ± 2.0 g) were collected from a local aquarium fish supplier in Bangi, Selangor, Malaysia and transported to the Marine Science laboratory of Universiti Kebangsaan Malaysia (UKM), Bangi, Selangor, Malaysia, where the experiment was conducted. After a week of acclimatization in controlled water temperature (26 °C), 180 individuals were indiscriminately divided into 12 recirculating aquaculture system (RAS) aquaria (size dimension and volume: 67 cm × 40 cm × 35 cm, 94 L; stocking density: 15 fish/tank, 0.02 kg/m^3^) equipped with their own submersible thermostat heaters (DoPhin 200 W, Penang, Malaysia). The fish were exposed to four experimental temperatures, 26 °C, 28 °C, 30 °C, and 32 °C, in triplicate by initiating a change of 1 °C/day. 

At the achieved temperature, fish were once again acclimatized for two days before the initial measurement of fish body weight (W_i_). Prior to taking measurements, fish were anesthetized with α-methyl quinoline (TransmoreR; Nika Trading, Puchong, Malaysia) following the method adopted by De et al. [18]. The 70-day experiment started immediately following the initial body weight measurement, between February and April 2020. Fish were fed twice daily (09:00 am and 16:00 pm) at the rate of 2% of mean body wet weight of fish. The dietary feeding rate of fish was adjusted weekly in accordance with the weight of growing fish. Uneaten pellets were collected for the calculation of feed utilization parameters. Other conditional factors such as water level (75% of the total volume of aquaria), pH level (pH 7.1), salinity (0.5 ppt), and photo-regimen (12 h light:12 h dark) were maintained throughout the study in order to preserve the integrity of data obtained in this study. 

### 2.2. Growth Performance and Gastric Emptying Time (GET) Evaluation

Following the initial weight measurement, fish were weighed on a weekly basis. Weight records were used to analyse growth performance in terms of:

Body weight gain (BWG, g) = (W_f_ − W_i_) × *n* [19],
Specific growth rate (SGR, % day^−1^) = (lnW_f_ − lnW_i_)/*t* × 100 [20],
Relative growth rate (RGR, %) = (W_f_ − W_i_)/W_i_ × 100 [21],
Daily growth rate (DGR, % day^−1^) = (W_f_ − W_i_)/*t* × 100 [8],
Survival (%) = *n*/initial number of fish stocked × 100 [22],
Food consumption (FC, g day^−1^) = food consumed in g × *t*^−1^ [23],
Food conversion ratio (FCR) = total feed fed/total weight gained [20],
Food conversion efficiency (FCE, %) = (Weight gain/Feed intake) × 100 [24]

Initial weight and final weight were indicated as W_i_ and W_f_, the total number of fish survived at the end of the experiment was indicated as *n*, and time (in days) was demonstrated as *t*.

Fish were returned to their respective experimental tanks after final weight measurement. Fish were starved for 72 h prior to GET evaluation. Following fish pellet preparation [15], fish were evaluated under a microradiographic unit (M60, Softex, Tokyo, Japan) at predetermined times since feeding; 0, 2, 4, 6, 8, 10, 12, 14, 16, and 18 h. Five fishes were selected from each treatment at each predetermined time. The anesthetizing method of De et al. [18] was also adopted for GET evaluation. Fish guts were photographed to trace the movement of food along the alimentary tract. Analysed fish were marked using the fin clipping method and released back to their respective tanks for recovery. 

### 2.3. Statistical Analysis

Statistical analyses performed in this study followed the methods outlined by Mazumder et al. [23]. All data obtained were analysed with one-way analysis of variance (ANOVA), and significant data (*p* < 0.05) were used for pairwise multiple comparisons with Tukey’s test using MINITAB version 19 (Minitab LLC, State College, PA, USA). A GET graph was generated using Microcal Origin 6.0 graphic software (OriginLab Corporation, Northampton, MA, USA).

## 3. Results and Remarks

### 3.1. Growth Performance of African Catfish (C. Gariepinus)

The present study revealed that the elevation of temperature from 26 to 32 °C did not lead to any significant difference (*p* > 0.05) in regard to growth parameters such as W_i_, W_f_, BWG, SGR, RGR, DGR, survival rate, and FC (Table 1). Initial weight (W_i_) in all treatments showed no significant difference (*p* > 0.05), reflecting on the homogeneity in fish weight at the beginning of the experiment. This complements several previous observations which agreed that the optimal temperature for the best growth of African catfish lies between 26 and 32 °C [20,21,25,26]. 

Despite no noticeable significant difference in the aforementioned parameters, variation in rearing temperature did influence both the FCR and FCE of the reared African catfish (*p* < 0.05, Table 1). Higher group temperatures (30 and 32 °C) displayed the best FCR and FCE. The reduced FCR at higher temperatures indicated that fish utilized feed very well [20], and there was an increase in protease activity and protein digestibility [27,28]. As temperature rose, so did appetite, leading FCE to increase [29]. Similar to our study, numerous other freshwater fish species displayed great improvement in FCR and FCE as temperature increased [29,30,31,32]. However, there were two major concerns that arose from the FCR and FCE acquired in our study; (1) the growth performance parameters observed in our study did not match the feed utilization data obtained (no significant difference in growth between treatments), and (2) the value of FCR obtained in our study was noticeably poorer as compared to FCR values obtained in other studies [20,33,34]. The calculations for both FCR and FCE were complicated by the fact that mortality (induced by cannibalism) occurred in all treatments except 26 °C throughout the experimentation period, resulting in data variability. The wide variation may also be due to the differences in the genetic background of fish stocks as well as variation in the feeding types and rates given to fish during the experimentation.

### 3.2. Gastric Emptying Time

**Figure 1:**
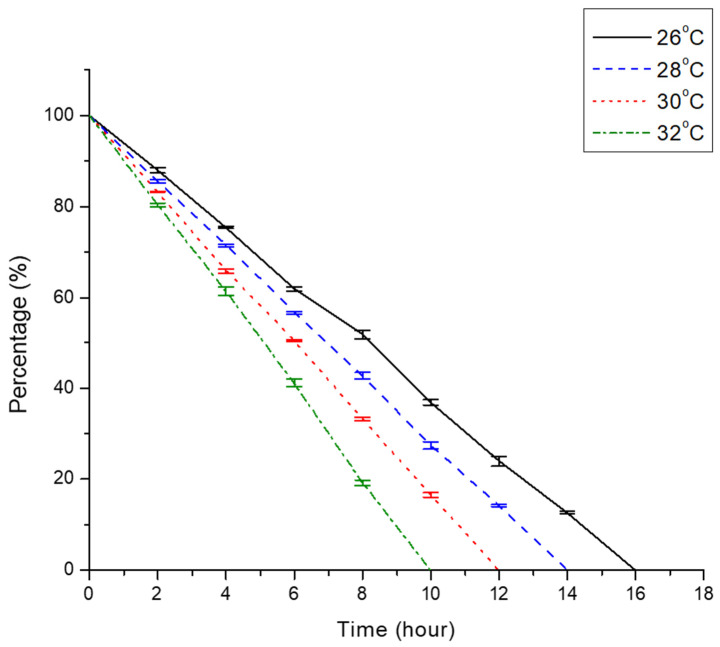
Percentage of feed retention in fish digestive system at an interval of two hours for each experimental group (26, 28, 30, and 32 °C). Error bars represent the SE of mean percentage values of five fish (*n* = 5) which were analysed at each time point.

From this study, we discovered that the GET of African catfish was extremely temperature dependent (Figure 1). The X-radiographic observations showed that as water temperature rose from 26 to 32 °C, the GET of African catfish shortened by an interval of two hours. The fastest emptying time was observed in group 32 °C, whereby fish feed was completely digested by the 10th hour. Group 30 °C exhibited a longer emptying rate, whereby fish feed was completely evacuated from fish guts by the 12th hour. Fish in group 28 °C took approximately 14 h in order for the fish feed to be completely evacuated from the alimentary tract. As compared to other experimental groups, the longest time taken (16 h) for fish feed to be completely evacuated from the digestive tract of fish was noted in group 26 °C.

At higher temperatures, the GET became shorter as there was an increase in fish digestive enzyme activity [35,36]. This finding also complements the assertion made by Suja et al. [37], whereby rapid digestion rate coincides with FCE. The time taken for African catfish to completely remove food items from the digestive tract was relatively different compared to other tropical fish species [8,10,23]. These differences were due to the types of feed given throughout the experimental period, feeding regime (e.g., duration and frequency), water temperature, and the fish species studied [23,38].

To summarize, water temperatures ranging from 26 to 32 °C had no significant effect on growth, although they did influence the GET of African catfish. To cover the gaps in this research area, more extensive physiological parameters could be considered. Here, we would also like to acknowledge that despite the substantial growth of African catfish farming today, parties involved in the culturing of this fish need effective containment and eradication plans in place to prevent escapees from harming the surrounding environment.

## Figures and Tables

**Table 1:** Growth performance and feed utilization parameters of *C. gariepinus* acclimated to 26, 28, 30, and 32 °C.

Parameters	26 °C	28 °C	30 °C	32 °C	*p*-Value
W_i_ (g)	18.14 ± 1.87	19.00 ± 1.22	20.33 ± 1.26	19.78 ± 0.79	0.26
W_f_ (g)	50.57 ± 4.12	50.31 ± 5.79	53.09 ± 5.21	49.18 ± 4.75	0.81
BWG (g)	486.43 ± 36.28	438.43 ± 64.51	458.61 ± 57.19	416.41 ± 56.00	0.82
SGR (% day^−1^)	1.50 ± 0.05	1.32 ± 0.07	1.30 ± 0.09	1.26 ± 0.08	0.10
RGR (%)	187.59 ± 10.50	155.86 ± 12.19	154.28 ± 14.50	146.16 ± 13.84	0.11
DGR (% day^−^^1^)	46.33 ± 3.46	44.74 ± 6.58	46.80 ± 5.83	42.49 ± 5.71	0.94
Survival (%)	100 ± 0.00	95.56 ± 2.22	95.56 ± 4.44	93.33 ± 3.85	0.72
FC (g day^−^^1^)	0.76 ± 0.06	0.77 ± 0.11	0.84 ± 0.10	0.85 ± 0.11	0.87
FCR	2.01 ± 0.03 ^a^	1.79 ± 0.03 ^ab^	1.72 ± 0.04 ^bc^	1.64 ± 0.02 ^c^	<0.05
FCE (%)	49.85 ± 0.68 ^a^	55.93 ± 0.80 ^b^	58.36 ± 1.39 ^bc^	61.10 ± 0.66 ^c^	<0.05

The values are shown in the form of mean ± SE (SE = standard error of mean values per treatment). Different letters across the same row indicate the presence of significance differences among the temperature groups (*p* < 0.05).

## Data Availability

The data presented in this study are available on fair request from the corresponding author.
